# Constructivist grounded theory and intersectionality in critical qualitative research: pathways to understanding reproductive injustice

**DOI:** 10.1590/1980-220X-REEUSP-2025-0331en

**Published:** 2026-07-17

**Authors:** Ariane Teixeira de Santana, Telmara Menezes Couto, Patricia Santos de Oliveira

**Affiliations:** 1 Universidade Federal da Bahia, Escola de Enfermagem, Salvador, BA, Brazil.

**Keywords:** Grounded Theory, Intersectional Framework, Critical Theory, Qualitative Research, Methods

## Abstract

**Objective:**

To reflect on the potential of Constructivist Grounded Theory articulated with intersectionality as a critical methodological and epistemological strategy in the investigation of reproductive injustices.

**Method:**

Theoretical, reflective study with a dialectical perspective, using bibliographic research as its investigative resource.

**Results:**

The articulation between the constructivist approach of Constructivist Grounded Theory and intersectionality makes it possible to understand phenomena such as obstetric racism based on the coconstruction of meanings, revealing how race, gender, and class operate relationally in the production of inequalities in reproductive care. This methodological-theoretical integration broadens the analytical scope of qualitative research by incorporating researcher reflexivity.

**Final considerations:**

The combination of these approaches strengthens the production of knowledge committed to social transformation, providing support for critical investigations in nursing and public health. Its applicability lies in the possibility of guiding research sensitive to structural inequalities, contributing to the formulation of more equitable reproductive care practices and policies.

## INTRODUCTION

Grounded Theory (GT) presents itself as a methodological possibility in qualitative research, combining guidelines for data collection and analysis. It is an inductive approach in which new theories regarding social processes are based on real experiences. Since its conception, GT has become a very powerful tool for the social sciences^(1,2)^.

GT was developed by two sociologists, Barney Glaser and Anselm Strauss, in the United States in the early 1960s. Initially, Glaser and Strauss collaborated on a research project regarding interaction between patients and healthcare professionals in hospitals, and their ideas were first published in *Discovery of Grounded Theory* in 1967. During this project, they developed a research methodology that became the basis of GT^(1,3)^. Of course, the method did not emerge solely from the work of Glaser and Strauss. If we examine previous work developed at the Chicago School, we can identify elements shared with other methods^(1)^.

Although Glaser and Strauss initially contributed jointly to the development of GT, over time they developed different strands for the application of the methodology^(3)^. These approaches present distinct philosophical assumptions that influence the ways their methods are understood and implemented, while preserving important seminal similarities. Understanding the similarities and differences among these approaches may facilitate the methodological pathway and determine the best fit for each study and each researcher’s worldview^(4)^.

Thus, the constructivist approach developed by sociologist Kathy Charmaz, who repositioned GT toward this stance through relativist ontology and subjective epistemology, is defined as the one that best fits the present study, which discusses reproductive injustice. Constructivist Grounded Theory (CGT) has a subjective epistemology, as it assumes that researchers are not separate from the research process and that knowledge is co-created^(3)^.

CGT values subjective experience and the meaning individuals attribute to their lived experiences. In contexts where healthcare reflects social inequalities, this strand offers an essentially humanizing approach. Rather than treating individual experiences as “objective” data, the constructivist researcher should understand these experiences as reflections of social, cultural, and institutional relationships^(2)^. In the context of reproductive justice, this perspective is fundamental because it helps consider that obstetric care does not occur in a vacuum, but rather within a network of social and structural norms that unequally affect individuals.

As a theoretical lens, intersectionality proposed by Kimberlé Crenshaw offers a framework for understanding how different forms of oppression, such as racism, sexism, and classism, interact and shape the experiences of marginalized people^(5)^. Reproductive health and obstetric care are areas in which these oppressions frequently manifest in an intersectional manner. Black and poor women, for example, face not only the sexism inherent in the denial of their reproductive choices, but also the racism that often classifies them as “less deserving” of respectful and qualified care.

Given these considerations, this essay proposes reflecting on how CGT can be used as a methodological tool based on the theoretical analytical framework of intersectionality. Thus, with the intention of critically contributing to the field, this text aims to reflect on the potential of CGT articulated with intersectionality as a critical methodological and epistemological tool in the investigation of reproductive injustices.

## METHOD

This is a theoretical study of a critical-reflective nature, grounded in conceptual analysis and epistemological problematization of national and international scientific productions concerning the dialectical reflection between CGT and inter-sectionality in the investigation of reproductive injustices.

The theoretical production was constructed through systematic reading of classical and contemporary works by means of a literature review in books and national and international databases such as Scopus, Web of Science, PubMed, and SciELO. Publications discussing the epistemological and methodological foundations of CGT, theoretical and empirical productions regarding intersectionality as a critical analytical tool, studies articulating qualitative research, social justice and health, as well as literature related to obstetric racism and reproductive inequalities were included.

The analysis was developed through critical and comparative reading of the selected works, identification of conceptual convergences and tensions, and construction of interpretative syntheses guided by the following analytical questions: how does this approach contribute to understanding reproductive injustices? In what way does the articulation between CGT and intersectionality broaden the critical potential of qualitative research?

The epistemological framework supporting this essay is grounded in the relativist ontology and subjective epistemology characteristic of the constructivist strand of GT, which understands knowledge as situated social construction produced through interaction among researcher, participants, and context^(2,3,6)^. This position is combined with intersectionality as a critical social theory that recognizes the interweaving of multiple systems of power, such as racism, patriarchy, and classism, in the production of structural inequalities^(7,8)^.

This methodological strategy not only enabled description of the analyzed frameworks, but also their critical interpretation, revealing their contributions, limitations, and possibilities of application in the field of reproductive health research and the confrontation of obstetric racism.

### Constructivist Grounded Theory and its Assumptions

Kathleen Marian Charmaz, born on August 19, 1939, in Whitehall, Wisconsin, died on July 27, 2020. She was an American sociologist belonging to the second generation of grounded theorists. Kathy Charmaz became an innovative and renowned theorist working in qualitative methodological approaches, developing the CGT method. Charmaz indicates that the researcher is not a neutral observer, but rather an active participant in knowledge construction, influenced by their values and experiences, contrary to the classical and objectivist view of Glaser and Strauss^(3,9)^.

The epistemological and ontological foundations of GT have changed over the years, especially with the challenges proposed by the constructivist strand^(1)^. CGT represents an external qualitative approach to constructing theory emerging from research data in interaction with the researcher. It is important to emphasize, however, that despite being a contemporary revision, it preserves the pragmatic foundations of GT proposed by Glaser and Strauss^(10,11)^.

The constructivist position by Charmaz presents relativist ontology and subjective epistemology, informing how knowledge production is socially produced and recognized from multiple points of view, both those of research participants and researchers. It proposes a reflexive stance regarding the data presented in the interaction between researcher and participants in the field setting for analytical constructions^(3)^. Thus, the researcher’s human situatedness in knowledge production becomes evident, as well as how both process and product are produced from these intersections.

Researchers using CGT may rely on several theoretical starting points to begin their investigations, such as feminist theory, post-structuralism, Marxist theory, critical theories, or Symbolic Interactionism itself. According to Charmaz, although Symbolic Interactionism and GT form a powerful “theorymethod package”, grounded theorists should not subscribe to orthodoxy between symbolic interactionism theory and GT. Thus, it is important to understand that GT may be used from other theoretical starting points or analytical repertoires to support the data^(3,12)^.

For Charmaz, knowledge rests on social constructions. Scholars using CGT should understand that knowledge is produced when dealing with empirical problems. Knowledge construction occurs under preexisting structural conditions and emerging situations, being influenced by researchers’ perspectives, privileges, social positions, interactions, and geographic locations. All these conditions are inherent to the research situation, yet they are often completely ignored or unmentioned in studies^(3,13)^.

CGT values the complexity and multiplicity of meanings presented in the data, since it is from them that a richer understanding of social characteristics is constructed. One example is the idea that social realities and constructed interpretations are not neutral, but shaped by the historical, cultural, and social contexts of both participants and researchers^(1,13)^.

By locating participants’ meanings and actions, the researcher reveals the connections between the micro and macro levels of analysis, linking, in this context, the subjective and social dimensions of the studied object. Participants’ meanings may reflect ideologies, actions, social conventions, or power relations. CGT seeks to analyze the assumptions upon which participants construct their meanings and actions. Charmaz viewed analysis in CGT as an interpretive representation rather than objective reports or a single point of view regarding the topic. As a result, the researcher sharpens awareness regarding the relativity of the data, which present multiple realities^(1,3)^.

The coding process in CGT is similar to that of Classical Grounded Theory, since it includes at least two phases—initial and focused coding—and is less prescriptive in its data analysis. Initial coding is the first phase of analysis; in it, the researcher explores the data in detail, line by line and incident by incident, to identify initial codes. At this stage, codes are created from what is happening in each segment of the data, allowing for open and flexible analysis^(3,10)^. For this stage, it is recommended that researchers use gerunds in the initial codes, as this helps capture the action, process, or meaning contained in participants’ statements. Rather than directly labeling the experience, the researcher attempts to interpret what is happening in the analyzed speeches, observations, or texts. These initial codes are often varied and may be adjusted and refined throughout the analysis^(3)^.

In the second phase, focused coding, the researcher reviews the initial codes and selects those that are more significant or recurrent in the data to generate provisional categories. Focused coding helps simplify and organize the data in a more grouped manner, contributing to the identification of broader categories and patterns^(3)^. This phase implies a higher level of interpretation, as the researcher begins comparing initial codes with one another and grouping the data into emerging categories. The objective is to identify codes that are more representative of the central specifics explaining patterns in the data. Thus, concepts are refined in relation to the data at a more abstract level, allowing a search for understanding the underlying connections and structures^(3)^.

Theoretical coding begins with focused coding and culminates in the final theory, aiming to integrate the categories and generate a substantive theory. At this stage, the researcher uses focused codes to develop a broader understanding in order to identify relationships and interactions among the main categories. Theoretical coding helps construct a theoretical narrative explaining the processes and patterns observed in the data^(3)^. CGT emphasizes that theoretical sensitivity is an interpretive process in which the researcher seeks to construct a theory faithful to the context and experiences of participants. The theoretical phase results in a more complete and in-depth analysis that connects and gives meaning to different aspects of the data^(11)^.

In all coding phases in CGT, reflexivity is important. The researcher must remain continuously aware of their own position, influences, and interpretations. This means considering their role in the construction of knowledge and recognizing that codes and categories are the result of active interpretation rather than a neutral or objective description of the data^(3)^.

Another important aspect in CGT is the use of memoranda (memos) and diagrams to support the consolidation process and assist in theoretical development. Memoranda are reflexive notes documenting ideas, questions, and insights during the research process, enabling the researcher to explore and reflect on interpretations emerging from the data. Diagrams, in turn, help visualize relationships among categories and facilitate the organization of ideas and theory construction^(3,10,11,12)^.

CGT precisely describes how the researcher’s inductive and abductive thinking occurs, this being an interactive stage of analysis that requires from the analyst a reasoning process allowing movement back and forth between data and conceptualization as a fundamental part of data analysis^(3)^.

### Intersectionality as A Critical Analytical Tool

In the 1990s, researcher and activist Kimberlé Crenshaw, together with several other researchers, defined the term “inter-sectionality” to designate the intersections among race, gender, class, and other axes of subordination^(5)^.

For Crenshaw, intersectionality seeks to bring together the structural and dynamic consequences of interaction between two or more axes of subordination by analyzing the interaction among racism, patriarchy, class exploitation, and other systems of oppression, exposing inequalities constitutive of society^(5,14,15,16)^.

According to Black feminist theorists, intersectionality assists in understanding the articulation of different social categories that interact and structure people’s lives in society, especially those of Black women. This subalternization reveals the oppression, discrimination, inequality, and social injustice experienced by these individuals. Intersectionality facilitates understanding of the interrelationships generating stereotypes and hierarchizing people within social constructions of power(5,16).

In Brazil, the roots of the construction of intersectionality as a theory are linked to social and academic movements that gained prominence from the 1990s onward. The Black movement played a fundamental role in this production by highlighting interactions among race, class, and gender in the experiences of Black women and marginalized individuals. Brazilian Black feminist thought, led by figures such as Lélia Gonzalez and Sueli Carneiro, was a major driving force behind intersectional thinking. These intellectuals raised, in an intersectional manner, considerations regarding the problems faced by the Black population, especially women^(17,18)^.

Thus, both Brazilian and North American intellectuals and activists were responsible for denouncing the insufficiency of hegemonic feminism which, by universalizing the experiences of middle-class White women, silenced the voices of Black and marginalized women. Simultaneously, they also criticized the Black movement for failing to recognize the specific gender oppressions experienced by Black women. From these tensions, Black feminism constructed its own theoretical and political body seeking to understand the interactions among different systems of domination^(15,17)^.

Intersectionality was forged to achieve a more appropriate understanding of complex social problems. In the field of social justice, it was proposed as a way of identifying racial and gender discrimination in order to more deeply understand how such discriminations operate together and impose limitations on Black women^(16)^.

In 1991, Crenshaw wrote the article *Mapping the Margins: Intersectionality, Identity Politics, and Violence against Women of Color*, in which she used the term to analyze the intersectional position of Black women and their structural marginalization^(15)^. From this work, the author established a solid theoretical basis for understanding intersectionality as a tool for analysis and critical praxis, weaving together contributions from Critical Race Theory and establishing the provisional concept of “intersectionality”. This significant work contributed to the development of the theory as a crucial approach in both social movements and academic research concerning race, gender, and class^(5,8)^.

According to Crenshaw’s thinking, studies seeking to understand Black women cannot use a single analytical category. For her, these experiences can only be understood by examining the multiple social identities these women carry. Thus, through this critical lens, it becomes possible to understand how multiple systems of power are intrinsic to the social experiences of these women. Intersectionality is an analytical framework describing how mutually constructed systems of power produce distinct social positions for each individual and group within them^(5)^.

In the conceptual construction of intersectionality, Crenshaw warns about the dangers of analytical invisibility that arise when attention focuses exclusively on only one social marker while neglecting others^(5,14)^. In light of this understanding, she presented the concepts of overinclusion and underinclusion in order to elucidate this invisibility. According to the author, approaches considering only one axis of inequality—whether race or gender—tend to camouflage the experiences of those located at the intersection of different systems of oppression.

Intersectionality, as a critical analytical tool, is not limited to recognizing only the oppressions imposed by multiple social identities, but also proposes appreciation of the resistance and empowerment strategies configured through the intersections of these identities. This is fundamental for developing strategies to fight oppression and promote social justice^(7,8,16,19)^.

To understand how these interactions occur, Patricia Hill Collins developed the concept of the “matrix of domination”. This concept helps analyze the intersections within a society’s power structure. According to Collins, the matrix is a multidimensional structure composed of various forms of oppression and discrimination that intertwine and reinforce one another. These forms of oppression include, among others, racism, sexism, classism, and homophobia^(5,7)^.

As a critical social theory, intersectionality is a counterhegemonic tool focused on constructing critical theoretical analysis by examining, from its assumptions, the understanding of marginalized people’s experiences and highlighting the voices and knowledge of these individuals for their social emancipation. As a reflexive practice, intersectionality seeks to transform existing power structures. By using an intersectional approach, the researcher must construct an analysis exposing dominant power structures while also seeking to promote greater inclusion and equity in society^(7)^.

In this sense, intersectionality becomes politically consolidated within the scope of critical perspectives in the social sciences, particularly in dialogue with feminist, anti-racist, and decolonial studies. Through these theoretical interlocutions, it establishes itself as a critical social theory capable of revealing the interconnections among different systems of power. On the epistemic level, intersectionality is configured as a fundamental analytical tool for understanding the historical and institutional processes that produce and reproduce social inequalities.

### Constructivist Grounded Theory in Critical Investigation in Social Justice Research

CGT, according to Charmaz’s precepts, develops as a field of critical investigation by breaking with positivist approaches and assuming that knowledge is constructed, situated, and influenced by power relations. This approach broadens the scope of GT^(2)^, allowing it to be used as a tool for questioning and social transformation, especially in contexts marked by inequalities, such as obstetric racism, gender violence, and reproductive injustices.

CGT is grounded in the understanding that reality and knowledge are social constructions. This means that constructs emerge from the interaction between researchers and participants within specific contexts and that there is no “objective truth” to be discovered. This epistemological stance favors critical research by allowing analysis of how discourses and practices produce and reproduce social inequalities^(2,3)^.

In contexts where oppression, discrimination, and inequality have become the status quo, it is necessary to question their causes. In this sense, Charmaz emphasizes the use of strategic questions such as “Why?” and “What is happening here?” These questions help researchers conduct a more critical assessment by situating participants within the social, cultural, temporal, and situational conditions in which they live. This helps recognize how structural conditions and positions affect both the researcher and the research process^(2,7)^.

In CGT, researchers are invited to situate themselves within what is happening in the research space, recognizing themselves as part of it and remaining flexible and attentive to empirical events, language, and meaning, while above all assuming moral responsibilities arising from the research process. This approach requires careful examination of how subsequent findings are related to these conditions. With emphasis on reflexivity, researchers examine not only who they are in relation to the research, but also how this dialectic is established^(2,20,21,22)^.

Researchers in CGT are not neutral. They are part of the construction of knowledge and, therefore, must adopt a reflexive stance based on the following questions: who am I? What is my social position? How do my beliefs and experiences affect data collection and analysis? This critical reflexivity strengthens research rigor and broadens ethical and political commitment^(2,3)^.

The constructivist perspective understands research as a political and ethical process. The objective is not only to understand, but also to intervene in unjust realities. By constructing theories based on the lived experiences of historically marginalized groups (such as Black women, Indigenous peoples, and transgender individuals), CGT reveals mechanisms of oppression and opens space for resistance^(2)^.

One of CGT’s greatest strengths is giving centrality to the experiences and narratives of oppressed subjects. This is especially important when studying themes such as obstetric racism, in which institutional silences and hegemonic discourses attempt to deny or minimize the problem. By listening carefully and with critical sensitivity to silenced voices, CGT adopts the epistemology of critical thought.

The constructivist approach is attentive to the power relations permeating the research process, both regarding participants’ experiences and researchers’ positions. This allows a critical reading of the institutional, cultural, and symbolic structures sustaining discriminatory practices^(2,3)^. CGT, when associated with frameworks such as intersectionality, helps reveal how class, race, gender, and other categories interact to produce inequalities.

According to Charmaz, as a critical investigation, CGT makes it possible to follow the complexity of social phenomena without reducing them to closed categories. Instead of “fitting” data into preexisting theories, it seeks to create sensitive, contextual, and dynamic theories constructed from participants’ meanings^(2)^. This openness is essential for capturing the effects of institutional racism, exclusionary public policies, and neglect regarding reproductive rights.

Research using CGT with a focus on social justice develops from relational ethics, recognizing that the relationship with participants is dialogical, respectful, and horizontal. Research is not conducted “about” subjects, but rather with them. This ethical positioning strengthens commitment to changes benefiting the involved groups. Such a stance favors identification, analysis, and questioning of the institutional, cultural, and symbolic structures maintaining social inequalities, revealing how healthcare, education, justice, or social assistance systems may reproduce racism, sexism, classism, and other forms of prejudice^(20,21,22)^.

CGT is grounded in its pragmatic heritage of addressing practical problems and promoting social justice^(23)^, emphasizing process design and fostering the creation of connections among events and situations, meanings and actions, individuals and social structures that may otherwise remain invisible.

This version of GT provides tools enabling researchers to deepen their understanding of the studied situations and offers the opportunity to analyze them from multiple perspectives. Thus, by conceptualizing what they have learned, constructivist grounded theorists may offer new critical understandings. For Charmaz, CGT calls not only for examination of research decisions, but also for examination of the moral and social commitment associated with what is being studied^(2)^.

### Constructivist Grounded Theory and Intersectional Analysis in The Investigation of Obstetric Racism

Research using CGT from a critical perspective refers to a specific body of knowledge that questions the oppressive effects of institutional power, knowledge, and social problems^(21,23)^. CGT, in a critical approach, expands GT by integrating participatory methods and incorporating goals of transformative social change^(20)^.

This approach represents a methodology sensitive to the complexity of social phenomena. By breaking with the original positivism of GT, it recognizes the subjective and contextual dimensions of the research process^(2,3)^. Such a perspective is especially relevant for investigating experiences marked by structural inequalities, such as obstetric racism, a specific form of violence affecting socially racialized bodies within reproductive healthcare.

Recent studies have demonstrated persistent racial disparities in maternal outcomes and in the quality of obstetric care, revealing greater exposure of Black women to precarious care^(24,25,26,27)^. However, a significant portion of these studies remains centered on describing inequalities or identifying isolated factors, without processually modeling how such disparities are produced in everyday institutional interactions^(24,25,27)^. The articulation between CGT and intersectionality offers a methodological pathway capable of filling this gap by transforming the problematization of inequalities into systematic theoretical construction.

CGT assumes that social reality is multiple, relational, and historically situated^(2,28,29)^. By valuing the complexity and multiplicity of meanings present in the data, CGT recognizes that interpretations are not neutral, but shaped by historical, cultural, and social contexts affecting both participants and researchers^(3,11,22,23)^. In the case of obstetric racism, women’s reported experiences do not emerge within a social vacuum. They are immersed in institutional traditions, biomedical ideologies, racial hierarchies, and gender norms structuring the field of reproductive healthcare. This indicates that CGT and intersectionality offer a methodological pathway capable of translating this scenario.

The operationalization of intersectionality in CGT occurs through processual coding. At this stage, the researcher examines the material line by line to capture actions and interactions. This strategy avoids premature classifications and allows observation of how race, gender, and class dynamically articulate within the care experience^(3,11,23,30)^. By privileging processes, the analysis prevents identities from becoming fixed as static categories and highlights the social production of inequalities.

In focused coding, the initial codes are compared and grouped into denser and more explanatory categories^(3,11)^. This movement reveals patterns crossing different narratives and not distributed homogeneously^(1,6,22)^. Processes of undercare, moral suspicion, or narrative delegitimization tend to affect women situated at specific intersections of race, gender, and class more intensely^(24,25,26,27)^. Constant comparison makes it possible to connect these regularities to the institutional structures organizing care, shifting the analysis from an exclusively interpersonal level toward understanding structural mechanisms.

Theoretical coding integrates these categories into an explanatory model^(3,11,28)^ that makes evident how biomedical discourses, care protocols, and social hierarchies jointly operate in producing obstetric racism^(26)^. In this process, intersectionality acts as a critical lens exposing the interweaving of racism, sexism, and classism, while CGT functions as an analytical technology organizing these elements into substantive theory. The analysis ceases to be merely descriptive and begins to model the institutional regimes sustaining undercare.

In all stages, reflexivity occupies a central place^(3,28,29)^. CGT requires researchers to recognize their social position, assumptions, and ethical-political implications in the construction of knowledge^(2,23)^. In the investigation of obstetric racism, this epistemological vigilance is indispensable for avoiding naturalization of inequalities or reproduction of institutional discourses minimizing racial violence. Emerging categories are understood as interpretive constructions anchored in historical contexts of oppression and resistance.

Thus, by locating the actions and meanings produced by women in their narratives, the researcher reveals the connections between the micro and macro levels of analysis. On the subjective level, what women feel, interpret, or name as injustice becomes articulated with the structural level—protocols, discourses, and institutional practices organizing care. In this sense, CGT is not limited to describing experiences, but seeks to analyze the implicit assumptions sustaining participants’ actions and interpretations. For Charmaz, analysis constitutes an interpretive representation of the studied reality rather than an objective report or definitive description of the phenomenon^(2,3,6,23)^. Such an understanding sharpens researchers’ awareness regarding the relativity of data and the existence of multiple coexisting realities, an essential condition for an intersectional approach.

Thus, the integration between CGT and intersectionality not only describes experiences of reproductive injustice, but also models the institutional regimes producing them. By transforming the intersectional lens into a concrete analytical procedure, CGT makes it possible to articulate micro and macro levels of analysis and construct substantive theory committed to exposing and confronting racialized inequalities in obstetric care.

## FINAL CONSIDERATIONS

As part of the understanding developed throughout this text, it may be inferred that GT, in its constructivist strand, and the theoretical framework of intersectionality may be articulated in a complementary way to analyze complex social phenomena, especially those marked by structural inequalities. Both approaches share the same commitment to attentive listening to lived experiences, recognition of the multiple dimensions of oppression, and production of knowledge committed to social transformation. This methodological-theoretical articulation allows research not only to present the investigated phenomenon, but also to contribute to the promotion of equity and social justice.

CGT is grounded in the principle that social meanings are constructed through interactions and sociocultural contexts. By valuing CGT precepts such as researcher reflexivity, dialogue with participants, and co-construction of meanings, a favorable environment is created for the application of intersectionality. This is because researchers do not position themselves as neutral or external subjects, but rather recognize their social position and their implication within the power relations that the research itself seeks to problematize.

Therefore, by integrating CGT and intersectionality, researchers adopt an ethical and political stance committed to valuing historically silenced voices and producing transformative knowledge. This alliance between theory and method enables research to move beyond denunciation, offering support for the formulation of fairer and more inclusive practices and policies in the field of reproductive healthcare. Thus, it constitutes an investigative strategy that directly dialogues with the principles of social justice by recognizing, understanding, and confronting the multiple forms of oppression structuring the lives of women and people with a uterus in situations of vulnerability.

## Data Availability

The entire dataset supporting the results of this study was published in the article itself.

## References

[1] Morse JM, Bowers BJ, Charmaz K, Clarke AE, Corbin J, Porr CJ (2021). Developing grounded theory: The second generation revisited..

[2] Charmaz K (2020). With constructivist grounded theory you can’t hide: social justice research and critical inquiry in the public sphere.. Qual Inq.

[3] Charmaz K (2009). A construção da teoria fundamentada: guia prático para análise qualitativa..

[4] Rieger KL (2019). Discriminating among grounded theory approaches.. Nurs Inq.

[5] Collins PH, Bilge S (2021). Interseccionalidade..

[6] Charmaz K, Thornberg R (2021). The pursuit of quality in grounded theory.. Qual Res Psychol.

[7] Collins PH (2022). Bem Mais que Ideias: a Interseccionalidade como Teoria Social Crítica..

[8] Collins PH (2024). Intersecções letais: raça, gênero e violência. Tradução de Heci Regina Candiani..

[9] Adele EC, Bryant A, Clarke AE (2022). Older, wiser, and much more daring: on Kathy Charmaz’s creative explosion c1995–2020.. Festschrift in honour of Kathy Charmaz. Bingley: Emerald Publishing Limited.

[10] Charmaz K, Denzin NK, Lincoln Y (2003). Strategies of qualitative inquiry..

[11] Charmaz K (2006). Constructing grounded theory: a practical guide through qualitative analysis..

[12] Charmaz K, Denzin NK, Lincoln YS (2005). The Sage handbook of qualitative research..

[13] Charmaz K, Denzin N, Lincoln Y (2011). The SAGE handbook of qualitative research..

[14] Crenshaw KW (1991). Mapping the margins: intersectionality, identity politics, and violence against women of color.. Stanford Law Rev.

[15] Hooks B (2019). Teoria feminista: da margem ao centro..

[16] Davis A (2016). Mulheres, raça e classe..

[17] Gonzalez L (2020). Por um feminismo afro-latino americano..

[18] Carneiro S (2011). Racismo, sexismo e desigualdade no Brasil..

[19] Hooks B (2020). Ensinando Pensamento Crítico: sabedoria prática..

[20] Hense C, McFerran KS (2016). Toward a critical grounded theory.. Qual Res J.

[21] Sawyer JM, Brady SR (2022). Applying critical grounded theory to community intervention development methodology: designing the critical difference engagement approach.. Int J Qual Methods.

[22] Timonen V, Foley G, Conlon C (2018). Challenges when using grounded theory: A pragmatic introduction to doing GT research.. Int J Qual Methods.

[23] Charmaz K (2016). The power of constructivist grounded theory for critical inquiry.. Qual Inq.

[24] Pereira GMV, Pimentel VM, Surita FG, Silva AD, Brito LGO (2022). Perceived racism or racial discrimination and the risk of adverse obstetric outcomes: a systematic review.. Sao Paulo Med J.

[25] Garcia LM (2023). Obstetric violence in the United States and other high-income countries: an integrative review.. Sex Reprod Health Matters.

[26] Santana AT, Couto TM, Lima KTRDS, Oliveira PS, Bomfim ANA, Almeida LCG (2024). Obstetric racism, a debate under construction in Brazil: perceptions of black women on obstetric violence.. Cien Saude Colet.

[27] Boakye PN, Prendergast N, Bandari B, Anane Brown E, Odutayo AA, Salami S (2023). Obstetric racism and perceived quality of maternity care in Canada: Voices of Black women.. Womens Health.

[28] Bobbink P, Larkin P, Probst S (2024). Application and challenges of using a Constructivist Grounded Theory methodology to address an undertheorized clinical challenge: A discussion paper.. Int J Nurs Stud Adv.

[29] Coçkun KA (2020). New explanation for the conflict between constructivist and objectivist grounded theory.. Int J Qual Methods.

[30] Metelski FK, Santos JLGD, Cechinel-Peiter C, Fabrizzio GC, Schmitt MD, Heilemann M (2021). Constructivist Grounded Theory: characteristics and operational aspects for nursing research.. Rev Esc Enferm USP.

