# Supplementary material for: Constructivist grounded theory and intersectionality in critical qualitative research: pathways to understanding reproductive injustice
Source: Rev Esc Enferm USP. 2026 Jul 17;60:e20250331. doi: 10.1590/1980-220X-REEUSP-2025-0331en (PMC13390995; doi:10.1590/1980-220X-REEUSP-2025-0331en)
Supplement: Translation

# Translation: Teoria fundamentada construtivista e interseccionalidade na pesquisa qualitativa crítica: caminhos para compreender a injustiça reprodutiva

**DOI:** 10.1590/1980-220X-REEUSP-2025-0331pt

**Authors:** Ariane Teixeira de Santana, Telmara Menezes Couto, Patricia Santos de Oliveira

**Affiliations:** 1 Universidade Federal da Bahia, Escola de Enfermagem, Salvador, BA, Brasil.

**Keywords:** Teoria Fundamentada, Enquadramento Interseccional, Teoria Crítica, Pesquisa Qualitativa, Métodos

## Abstract

**Objetivo:**

Refletir sobre o potencial da Teoria Fundamentada Construtivista articulada à interseccionalidade como estratégia metodológica e epistemológica crítica na investigação de injustiças reprodutivas.

**Método:**

Estudo teórico, reflexivo, de perspectiva dialética, que tem na pesquisa bibliográfica seu recurso de investigação.

**Resultados:**

A articulação entre a abordagem construtivista da Teoria Fundamentada Construtivista e a interseccionalidade possibilita compreender fenômenos como o racismo obstétrico a partir da coconstrução de significados evidenciados de como a raça, o gênero e a classe operam de forma relacional na produção de desigualdades no cuidado reprodutivo. Essa integração metodológicoteórica amplia o alcance analítico da pesquisa qualitativa ao incorporar a reflexividade do(a) pesquisador(a).

**Considerações finais:**

a combinação dessas abordagens fortalece a produção de conhecimento comprometido com a transformação social, oferecendo subsídios para investigações críticas em enfermagem e saúde coletiva. Sua aplicabilidade reside na possibilidade de orientar pesquisas sensíveis às desigualdades estruturais, contribuindo para a formulação de práticas e políticas de cuidado reprodutivo mais equitativas.

## INTRODUÇÃO

A Teoria Fundamentada nos Dados (TFD) apresenta-se como uma possibilidade metodológica na pesquisa qualitativa, aliando diretrizes para a coleta e a análise de dados. Ela é uma abordagem indutiva, na qual novas teorias sobre processos sociais são baseadas em experiências reais. Desde a sua concepção, a TFD tornou-se uma ferramenta muito poderosa para as ciências sociais^(1,2)^.

A TFD foi desenvolvida por dois sociólogos, Barney Glaser e Anselm Strauss, nos Estados Unidos, no início dos anos 1960. Inicialmente, Glaser e Strauss colaboraram em um projeto de pesquisa sobre a interação entre pacientes e profissionais de saúde em hospitais, e suas ideias foram publicadas pela primeira vez em *Discovery of Grounded Theory*, em 1967. Durante esse projeto, eles desenvolveram uma metodologia de pesquisa que se tornou a base da TFD^(1,3)^. É claro que o método não surgiu apenas do trabalho de Glaser e Strauss. Se olharmos para o trabalho anterior desenvolvido na Escola de Chicago, veremos elementos em comum com outros métodos^(1)^.

Apesar de Glaser e Strauss terem contribuído inicialmente para o desenvolvimento da TFD, ao longo do tempo, eles desenvolveram vertentes diferentes para a aplicação da metodologia^(3)^. Essas abordagens apresentam diferentes suposições filosóficas que influenciam as maneiras pelas quais seus métodos são compreendidos e implementados, mas preservam semelhanças seminais importantes. Compreender as semelhanças e as diferenças presentes nessas abordagens pode facilitar o percurso metodológico e determinar a melhor adequação ao estudo e à visão de mundo de cada pesquisador(a)^(4)^.

Desse modo, a abordagem construtivista, desenvolvida pela socióloga Kathy Charmaz, que reposicionou a TFD para tal postura com a ontologia relativista e a epistemologia subjetiva, define-se como a que melhor se ajusta ao presente estudo, que discorre sobre injustiça reprodutiva. A Teoria Fundamentada Construtivista (TFC) tem uma epistemologia subjetiva, pois assume que os(as) pesquisadores(as) não estão separados(as) da pesquisa e que o conhecimento é cocriado^(3)^.

A TFC valoriza a experiência subjetiva e o significado que os indivíduos atribuem às suas vivências. Em contextos em que o atendimento de saúde reflete desigualdades sociais, essa vertente oferece uma abordagem essencialmente humanizadora. Ao invés de tratar as experiências individuais como dados “objetivos”, o(a) pesquisador(a) construtivista deve compreender essas vivências como reflexos de relações sociais, culturais e institucionais^(2)^. No contexto da justiça reprodutiva, essa perspectiva é fundamental, pois ajuda a considerar que a assistência obstétrica não ocorre em um vácuo, mas dentro de uma rede de normas sociais e estruturais que afetam desigualmente os indivíduos.

Como lente teórica, a interseccionalidade proposta por Kimberlé Crenshaw oferece um quadro para compreender como diferentes formas de opressão, como racismo, sexismo e classismo, interagem e moldam as experiências de pessoas marginalizadas^(5)^. A saúde reprodutiva e o atendimento obstétrico são áreas nas quais essas opressões frequentemente se manifestam de forma interseccional. Mulheres negras e pobres, por exemplo, enfrentam não só o sexismo inerente à negação de suas escolhas reprodutivas, mas também o racismo que as classificam, muitas vezes, como “menos merecedoras” de um atendimento respeitoso e qualificado.

Diante de tais ponderações, este ensaio se propõe a refletir como a TFC pode ser utilizada como ferramenta metodológica, a partir do cabedal analítico teórico interseccional. Desse modo, no intuito de contribuir criticamente, este texto objetiva refletir sobre o potencial da TFC articulada à interseccionalidade como ferramenta metodológica e epistemológica crítica na investigação de injustiças reprodutivas.

## MÉTODO

Trata-se de estudo teórico de natureza crítico-reflexiva, fundamentado na análise conceitual e na problematização epistemológica de produções científicas nacionais e internacionais sobre a reflexão dialética entre a TFC e a interseccionalidade na investigação de injustiças reprodutivas.

A produção teórica foi construída a partir da leitura sistemática de obras clássicas e contemporâneas, mediante revisão da literatura em livros e em bases de dados nacionais e internacionais, como Scopus, *Web of Science*, PubMed e SciELO. Incluíram-se publicações que discutem os fundamentos epistemológicos e metodológicos da TFC, produções teóricas e empíricas sobre interseccionalidade como ferramenta analítica crítica, estudos que articulam pesquisa qualitativa, justiça social e saúde, e literatura relacionada ao racismo obstétrico e às desigualdades reprodutivas.

A análise foi desenvolvida por meio de leitura crítica e comparativa das obras selecionadas, identificação de convergências e tensões conceituais e construção de sínteses interpretativas orientadas pelas perguntas analíticas: como essa abordagem contribui para a compreensão de injustiças reprodutivas? De que modo a articulação entre a TFC e a interseccionalidade amplia o potencial crítico da pesquisa qualitativa?

O referencial epistemológico que sustenta este ensaio ancorase na ontologia relativista e na epistemologia subjetiva, próprias da vertente construtivista da TFD, que compreendem o conhecimento como construção social situada, produzida na interação entre pesquisador(a), participantes e contexto^(2,3,6)^. Conjuga-se a esse posicionamento a interseccionalidade enquanto teoria social crítica, que reconhece a imbricação entre múltiplos sistemas de poder, como racismo, patriarcado e classismo, na produção de desigualdades estruturais^(7,8)^.

Essa estratégia metodológica não apenas possibilitou a descrição dos referenciais analisados, como também sua interpretação crítica, revelando suas contribuições, limites e possibilidades de aplicação no campo da pesquisa em saúde reprodutiva e enfrentamento ao racismo obstétrico.

### Teoria Fundamentada Construtivista e Seus Pressupostos

Kathleen Marian Charmaz, nascida em 19 de agosto de 1939 em Whitehall, Wisconsin, faleceu em 27 de julho de 2020. Foi uma socióloga norte-americana da segunda geração de teóricos fundamentados. Kathy Charmaz se tornou uma inovadora e renomada teórica atuante em abordagem metodológica qualitativa, desenvolvendo o método da TFC. Charmaz indica que o(a) pesquisador(a) não é um(a) observador(a) neutro(a), mas um(a) participante ativo(a) na construção do conhecimento, influenciado(a) por seus valores e experiências, ao contrário da visão clássica e objetivista de Glaser e Strauss^(3,9)^.

As bases epistemológicas e ontológicas da TFD mudaram ao longo dos anos, especialmente com os desafios propostos pela vertente construtivista^(1)^. A TFC representa uma abordagem qualitativa externa para a construção de uma teoria que emerge dos dados de pesquisa em interação com o(a) pesquisador(a). É importante ressaltar, contudo, que apesar de ser uma revisão contemporânea, ela preserva os fundamentos pragmáticos da TFD proposta por Glaser e Strauss^(10,11)^.

A posição construtivista assumida por Charmaz apresenta ontologia relativista e epistemologia subjetiva, e isso informa como a produção de conhecimento é um elemento produzido socialmente e reconhecido por múltiplos pontos de vista, tanto do(a) participante da pesquisa quanto do(a) pesquisador(a). Propõe uma postura reflexiva em relação aos dados apresentados na interação do(a) pesquisador(a) com os(as) participantes no cenário de campo para construções analíticas^(3)^. Observa-se, assim, a localidade humana do(a) pesquisador(a) na produção da pesquisa e como o processo e o produto são produzidos dessas interseções.

Os(as) pesquisadores(as) que utilizam a TFC podem se guiar por diversos pontos de partida teóricos para iniciar a investigação, como a teoria feminista, o pós-estruturalismo, a teoria marxista, as teorias críticas ou o próprio Interacionismo Simbólico. De acordo com Charmaz, apesar de o Interacionismo Simbólico e a TFD formarem um poderoso “pacote teoriamétodos”, o(a) pesquisador(a) fundamentado não deve subscrever uma ortodoxia entre o teórico interacionismo simbólico e a TFD. Assim, é importante compreender que a TFD pode ser usada a partir de outros pontos de partida teóricos ou repertório analítico para amparar os dados^(3,12)^.

Para Charmaz, o conhecimento repousa em construções sociais. Os estudiosos que utilizam a TFC devem compreender que o conhecimento é produzido quando se lida com problemas empíricos. A construção de conhecimento ocorre sob condições estruturais preexistentes e situações emergentes, sofrendo influência de perspectivas, privilégios, posições sociais, interações e localizações geográficas do(a) pesquisador(a). Todas essas condições são inerentes à situação de pesquisa, mas são completamente ignoradas ou não mencionadas pelos estudos^(3,13)^.

A TFC valoriza a complexidade e a multiplicidade de significados apresentados nos dados, pois é a partir deles que se constrói uma compreensão mais rica das características sociais. Um exemplo é a ideia de que as realidades sociais e as interpretações construídas não são neutras, mas moldadas pelos contextos históricos, culturais e sociais, tanto dos(as) participantes quanto dos(as) pesquisadores(as)^(1,13)^.

Ao localizar os significados e ações dos(as) participantes, o(a) pesquisador(a) revela as conexões entre os níveis micro e macro de análise, o que vincula, nesse contexto, o plano subjetivo e o social do objeto estudado. Os significados dos(as) participantes podem refletir ideologias, ações, convenções sociais ou relações de poder. A TFC procura analisar as suposições a partir das quais os(as) participantes constroem seus significados e ações. Charmaz via a análise na TFC como representação interpretativa, não como relatórios objetivos ou um único ponto de vista sobre o tópico. Como resultado, o(a) pesquisador(a) aguça a consciência para a relatividade dos dados, que apresenta suas múltiplas realidades^(1,3)^.

O processo de codificação da TFC é semelhante da Teoria Fundamentada Clássica, pois ela possui ao menos duas fases, codificação inicial e focalizada, e se apresenta menos prescritiva na sua análise de dados. A codificação inicial é a primeira fase de análise; nela, o(a) pesquisador(a) explora os dados de maneira detalhada, linha por linha, incidente por incidente, para identificar os códigos iniciais. Nesse estágio, os códigos são criados a partir do que está acontecendo em cada segmento dos dados, o que permite uma análise aberta e flexível^(3,10)^. Para essa etapa, é recomendado que os(as) pesquisadores(as) usem verbos no gerúndio para os códigos iniciais, pois isso ajuda a capturar a ação, o processo ou o significado contido nas falas dos(as) participantes. Ao invés de rotular a experiência diretamente, o(a) pesquisador(a) tenta interpretar o que está acontecendo nas falas, observações ou textos analisados. Esses códigos iniciais são frequentemente variados e podem ser ajustados e refinados durante a análise^(3)^.

Na segunda fase, a codificação focalizada, o(a) pesquisador(a) revisa os códigos iniciais e seleciona aqueles que são mais significativos ou recorrentes nos dados para gerar categorias provisórias. A codificação focalizada ajuda a simplificar e organizar os dados de forma mais agrupada, o que contribui para a identificação das categorias e padrões mais amplos^(3)^. Essa fase implica um nível mais alto de interpretação, pois o(a) pesquisador(a) começa a comparar os códigos iniciais entre si e a agrupar os dados em categorias emergentes. O objetivo é identificar códigos que sejam mais representativos dos específicos centrais para explicar padrões nos dados. Assim, refinam-se os conceitos em relação aos dados de forma mais abstrata, o que permite uma busca para entender as conexões e estruturas subjacentes^(3)^.

A codificação teórica começa com a codificação focalizada e culmina na teoria final, tendo como objetivo integrar as categorias e gerar uma teoria substantiva. Nessa etapa, o(a) pesquisador(a) utiliza os códigos focados para desenvolver uma compreensão mais ampla, a fim de identificar relações e interações entre as principais categorias. A codificação teórica ajuda a construir uma narrativa teórica que explica os processos e padrões observados nos dados^(3)^. A TFC enfatiza que a convicção teórica é um processo interpretativo no qual o(a) pesquisador(a) busca construir uma teoria que seja fiel ao contexto e às experiências dos(as) participantes. A fase teórica resulta em uma análise mais completa e profunda, que conecta e dá sentido a diferentes aspectos dos dados^(11)^.

Em todas as fases de codificação na TFC, a reflexividade é importante. O(a) pesquisador(a) deve estar continuamente consciente de sua própria posição, influências e interpretações. Isso significa considerar seu papel na construção do conhecimento ao perceber que os códigos e as categorias são resultados de uma interpretação ativa, e não uma descrição neutra ou objetiva dos dados^(3)^.

Outro aspecto importante na TFC é o uso de memorandos (memos) e diagramas para apoiar o processo de consolidação e ajudar no desenvolvimento teórico. Memorandos são anotações reflexivas que documentam ideias, questionamentos e *insights* durante a realização da pesquisa, o que possibilita que o(a) pesquisador(a) explore e reflita sobre as interpretações que emergem dos dados. Os diagramas, por sua vez, ajudam a visualizar as relações entre as categorias e facilitam a organização das ideias e a construção da teoria^(3,10,11,12)^.

A TFC descreve, de forma precisa, como se dá o pensamento indutivo e abdutivo do(a) pesquisador(a), sendo essa uma etapa interativa da análise que vai requerer do(a) analista um raciocínio que permita ir e voltar entre dados e conceituação como uma parte fundamental da análise de dados^(3)^.

### Interseccionalidade como Ferramenta de Analítica Crítica

Na década de 1990, a pesquisadora e ativista Kimberlé Crenshaw, juntamente com diversas outras pesquisadoras, definiu o termo “interseccionalidade” para designar as interseções entre raça, gênero, classe e outros eixos de subordinação^(5)^.

Para Crenshaw, a interseccionalidade busca conjugar consequências estruturais e dinâmicas da interação entre dois ou mais eixos da subordinação ao analisar a interação entre racismo, patriarcado, exploração de classe e outros sistemas de opressão, expondo desigualdades constitutivas da sociedade^(5,14,15,16)^.

Segundo teóricas do feminismo negro, a interseccionalidade auxilia na compreensão da articulação de diferentes categorias sociais que se articulam e estruturam a vida das pessoas em sociedade, em especial de mulheres negras. Essa subalternização evidencia a opressão, a discriminação, a desigualdade e a injustiça social que essas pessoas experienciam. A interseccionalidade facilita a visão sobre as interrelações que geram estereótipos e hierarquizam pessoas dentro de construções sociais de poder^(5,16)^.

No Brasil, as raízes para a construção da interseccionalidade como teoria estão ligadas a movimentos sociais e acadêmicos que ganharam destaque a partir da década de 1990. O movimento negro desempenhou um papel fundamental nessa produção, ao destacar as interações entre raça, classe e gênero na experiência das mulheres negras e pessoas marginalizadas. O pensamento feminista negro brasileiro, liderado por uras como Lélia Gonzalez e Sueli Carneiro, foi um grande impulsionador do pensamento interseccional. Essas intelectuais apontavam, de forma interseccional, considerações a respeito dos problemas enfrentados pela população negra, especialmente pelas mulheres^(17,18)^.

Desse modo, tanto as intelectuais e ativistas brasileiras quanto as norte-americanas foram responsáveis por denunciar a insuficiência de um feminismo hegemônico que, ao universalizar a experiência das mulheres brancas de classe média, silenciava as vozes das mulheres negras e marginalizadas. Paralelamente, também criticavam o movimento negro por não reconhecerem as especificidades das opressões de gênero vividas por mulheres negras. A partir dessas tensões, o feminismo negro construiu um corpo teórico e político próprio, que buscava compreender as interações entre diferentes sistemas de dominação^(15,17)^.

Para uma compreensão mais apropriada de problemas sociais complexos, a interseccionalidade é forjada. No campo da justiça social, propôs-se identificar a discriminação racial e a discriminação de gênero para compreender mais profundamente como tais discriminações operam juntas e impõem limitações para as mulheres negras^(16)^.

Crenshaw, em 1991, escreveu o artigo “*Mapping the Margins: Intersectionality, Identity Politics, and Violence against Women of Color*”, e nele, ela usa o termo para analisar a posição interseccional das mulheres negras e sua marginalização estrutural^(15)^. A partir desse trabalho, a autora concebe uma base teórica sólida para compreender a interseccionalidade como uma ferramenta de análise e *práxis* crítica, tecendo aportes da teoria crítica de raça e estabelecendo o conceito provisório sobre a “interseccionalidade”. Essa obra significativa contribuiu para o desenvolvimento da teoria como um enfoque crucial tanto nos movimentos sociais quanto na pesquisa acadêmica sobre raça, gênero e classe^(5,8)^.

De acordo com o pensamento de Crenshaw, os estudos que buscam compreender as mulheres negras não podem utilizar uma categoria de análise única. Para ela, essas experiências só podem ser compreendidas quando se examinam as múltiplas identidades sociais que essas mulheres carregam. Assim, a partir dessa lente crítica, poderemos enxergar como os múltiplos sistemas de poder são intrínsecos na vivência social dessas mulheres. A interseccionalidade é uma base analítica que descreve como sistemas de poder que se constroem mutuamente e produzem lugares sociais distintos para cada indivíduo e grupo dentro deles^(5)^.

Na construção conceitual da interseccionalidade, Crenshaw sinaliza sobre os perigos da invisibilidade analítica que surgem quando um olhar se concentra exclusivamente em um marcador social apenas, negligenciando os outros^(5,14)^. Com vistas a essa compreensão, ela apresentou os conceitos de superinclusão e subinclusão, na busca por elucidar essa invisibilidade. Segundo a autora, abordagens que consideram apenas um eixo de desigualdade, seja raça ou gênero, tendem a camuflar as experiências daquelas que se encontram na intersecção entre diferentes sistemas de opressão.

A interseccionalidade, como ferramenta de análise crítica, não se limita a reconhecer apenas as opressões que as múltiplas identidades sociais imprimem, mas se propõe a apreciar as estratégias de resistência e empoderamento que se conuram das interseções dessas identidades. Isso é fundamental para desenvolver estratégias de luta contra a opressão e promover a justiça _social_(7,8,16,19)_._

Para compreender como essas interações acontecem, Collins desenvolveu o conceito de “matriz de dominação”. Ele ajuda a analisar as interseções entre a estrutura de poder de uma sociedade. Para ela, a matriz é uma estrutura multidimensional composta por várias formas de opressão e discriminação que se entrelaçam e se reforçam mutuamente. Essas formas de opressão incluem, entre outras, o racismo, o sexismo, o classismo e a homofobia^(5,7)^.

Como teoria social crítica, a interseccionalidade é uma ferramenta contra-hegemônica que se debruça sobre a construção de uma análise teórica crítica ao examinar, a partir de seus pressupostos, a compreensão das experiências de pessoas marginalizadas e ao ressaltar as vozes e os conhecimentos desses indivíduos para sua emancipação social. Como prática reflexiva, a interseccionalidade busca transformar as estruturas de poder existentes. Ao utilizar uma abordagem interseccional, o(a) pesquisador(a) deve construir uma análise que exponha as estruturas de poder dominante, além de buscar promover uma maior inclusão e equidade na sociedade^(7)^.

Nesse sentido, a interseccionalidade consolida-se politicamente no âmbito das perspectivas críticas das ciências sociais, particularmente em diálogo com os estudos feministas, antirracistas e decoloniais. A partir dessas interlocuções teóricas, ela se afirma como uma teoria social crítica capaz de revelar as interconexões entre diferentes sistemas de poder. No plano epistêmico, a interseccionalidade se conura como uma ferramenta analítica fundamental para compreender os processos históricos e institucionais que produzem e reproduzem desigualdades sociais.

### Teoria Fundamentada Construtivista na Investigação Crítica em Pesquisa de Justiça Social

A TFC, segundo os preceitos de Charmaz, desenvolve-se como um campo de investigação crítica ao romper com abordagens positivistas e assumir que o conhecimento é construído, situado e influenciado por relações de poder. Essa abordagem amplia o alcance da TFD^(2)^, permitindo que ela seja usada como ferramenta de questionamento e transformação social, especialmente em contextos marcados por desigualdades, como no racismo obstétrico, na violência de gênero e nas injustiças reprodutivas.

A TFC parte do entendimento de que realidade e o conhecimento são construções sociais. Isso significa que os construtos emergem da interação entre pesquisadores(as) e participantes, em contextos específicos, e que não há uma “verdade objetiva” a ser descoberta. Essa postura epistemológica favorece uma pesquisa crítica ao permitir que se analisem como os discursos e as práticas produzem e reproduzem desigualdades sociais^(2,3)^.

Nos contextos em que a opressão, a discriminação e a desigualdade se tornaram o *status quo,* é preciso questionar as causas. Nesse sentido, Charmaz orienta a ênfase na utilização de perguntas estratégicas como “Por quê?” e “O que está acontecendo aqui?”. Essas perguntas ajudam o(a) pesquisador(a) a realizar uma avaliação mais crítica, ao localizar os(as) participantes da pesquisa dentro das condições sociais, culturais, temporais e situacionais em que eles vivem. Isso ajuda a reconhecer como as condições e posições estruturais afetam o(a) pesquisador(a) e o processo de pesquisa^(2,7)^.

Na TFC, os(as) pesquisadores(as) são convidados(as) a se situar no que está acontecendo no espaço da pesquisa, reconhecendo-se como parte dela e permanecendo flexíveis e vigilantes aos eventos empíricos, à linguagem e ao significado, assumindo, sobretudo, responsabilidades morais decorrentes da pesquisa. Essa abordagem exige um exame minucioso de como as descobertas subsequentes estão relacionadas a essas condições. Com ênfase na reflexividade, não apenas se examina quem se é em relação à pesquisa, mas também como essa dialética se estabelece^(2,20,21,22)^.

O(a) pesquisador(a) na TFC não é neutro(a). Ele(a) é parte da construção do conhecimento, e, por isso, é necessário adotar uma postura reflexiva a partir dos seguintes questionamentos: quem sou eu? Qual meu lugar social? Como minhas crenças e experiências afetam a coleta e análise de dados? Essa reflexividade crítica fortalece o rigor da pesquisa e amplia o compromisso ético e político^(2,3)^.

A perspectiva construtivista entende a pesquisa como um processo político e ético. O objetivo não é apenas compreender, mas também intervir nas realidades injustas. Ao construir teorias, a partir da experiência vivida de grupos historicamente marginalizados (como mulheres negras, indígenas, pessoas trans), a TFC revela mecanismos de opressão e abre espaço para resistências^(2)^.

Um dos grandes trunfos da TFC é dar centralidade às experiências e às narrativas de sujeitos oprimidos. Isso é especialmente importante ao se estudar temas como o racismo obstétrico, em que silêncios institucionais e discursos hegemônicos tentam negar ou minimizar o problema. Ao ouvir atentamente e com sensibilidade crítica as vozes silenciadas, a TFC adota a epistemologia do pensamento crítico.

A abordagem construtivista está atenta às relações de poder que atravessam o processo de pesquisa tanto no que tange às experiências dos(as) participantes quanto à postura dos(as) pesquisadores(as). Isso permite uma leitura crítica das estruturas institucionais, culturais e simbólicas que sustentam práticas discriminatórias^(2,3)^. A TFC, quando associada a referenciais como a interseccionalidade, ajuda a revelar como classe, raça, gênero e outras categorias interagem para produzir desigualdades.

Segundo Charmaz, como investigação crítica, a TFC permite acompanhar a complexidade dos fenômenos sociais sem reduzi-los a categorias fechadas. Em vez de “encaixar” os dados em teorias pré-existentes, ela busca criar teorias sensíveis, contextuais e dinâmicas, construídas com base nos significados dos(as) participantes^(2)^. Essa abertura é essencial para captar os efeitos do racismo institucional, das políticas públicas excludentes e da negligência com os direitos reprodutivos.

Pesquisas que utilizam a TFC com foco na justiça social desenvolvem-se a partir da ética relacional, reconhecendo que a analogia com os(as) participantes é dialógica, respeitosa e horizontal. A pesquisa não se faz “sobre” os sujeitos, mas com eles. Esse posicionamento ético fortalece o compromisso com mudanças que beneficiem os grupos envolvidos. Essa postura favorece identificar, analisar e questionar as estruturas institucionais, culturais e simbólicas que mantêm as desigualdades sociais, evidenciando como os sistemas de saúde, educação, justiça ou assistência social podem reproduzir racismos, sexismos, classismos e outros preconceitos^(20,21,22)^.

A TFC fundamenta-se em sua herança pragmática de atender a problemas práticos e promover a justiça social^(23)^, ao enfatizar o desenho de processos e promover a criação de conexões entre eventos e situações, significados e ações, indivíduos e estruturas sociais que podem permanecer invisíveis.

Essa versão da TFD fornece ferramentas que permitem que os(as) pesquisadores(as) tenham um aprofundamento nas situações estudadas e proporciona a oportunidade de analisá-las a partir de vários pontos de vista. Assim, ao conceituar o que aprendeu, os teóricos fundamentados construtivistas podem oferecer novos entendimentos críticos. Para Charmaz, a TFC convoca a examinar não apenas as decisões de pesquisa, mas também a examinar o compromisso moral e social junto ao que se estuda^(2)^.

### Teoria Fundamentada Construtivista e Análise Interseccional na Investigação do Racismo Obstétrico

Pesquisas que utilizam a TFC na perspectiva crítica apresentam referência a um corpo específico de conhecimento que questiona os efeitos opressivos do poder institucional, do conhecimento e dos problemas sociais^(21,23)^. A TFC, em uma abordagem crítica, expande a TFC, integrando métodos participativos e incorporando metas de mudança social transformadora^(20)^.

Essa abordagem representa uma metodologia sensível à complexidade dos fenômenos sociais. Ao romper com o positivismo original da TFD, ela reconhece as dimensões subjetivas e contextuais do processo de pesquisa^(2,3)^. Tal perspectiva é especialmente relevante para investigar experiências marcadas por desigualdades estruturais, como é o caso do racismo obstétrico, uma forma específica de violência que incide sobre corpos socialmente racializados no campo da saúde reprodutiva.

Pesquisas recentes têm demonstrado disparidades raciais persistentes nos desfechos maternos e na qualidade do cuidado obstétrico, o que evidencia maior exposição de mulheres negras a cuidados precarizados^(24,25,26,27)^. Entretanto, parte significativa desses estudos permanece centrada na descrição das desigualdades ou na identificação de fatores isolados, sem modelar processualmente como tais disparidades se produzem nas interações institucionais cotidianas^(24,25,27)^. A articulação entre TFC e interseccionalidade oferece um caminho metodológico capaz de preencher essa lacuna ao transformar a problematização das desigualdades em construção teórica sistemática.

A TFC parte do pressuposto de que a realidade social é múltipla, relacional e historicamente situada^(2,28,29)^. Ao valorizar a complexidade e a multiplicidade dos significados presentes nos dados, a TFC reconhece que as interpretações não são neutras, mas moldadas por contextos históricos, culturais e sociais que atravessam tanto os(as) participantes quanto os(as) pesquisadores(as)^(3,11,22,23)^. No caso do racismo obstétrico, as experiências relatadas pelas mulheres não emergem em um vazio social. Elas estão imersas em tradições institucionais, ideologias biomédicas, hierarquias raciais e normas de gênero que estruturam o campo da saúde reprodutiva. Isso indica que a TFC e a interseccionalidade oferecem um caminho metodológico capaz de traduzir esse cenário.

A operacionalização da interseccionalidade na TFC ocorre por meio da codificação processual. Nessa etapa, o(a) pesquisador(a) examina o material linha por linha para capturar ações e interações. Essa estratégia evita classificações prematuras e permite observar como raça, gênero e classe se articulam dinamicamente na experiência do cuidado^(3,11,23,30)^. Ao privilegiar processos, a análise impede a fixação de identidades como categorias estáticas e evidencia a produção social das desigualdades.

Na codificação focalizada, os códigos iniciais são comparados e agrupados em categorias mais densas e explicativas^(3,11)^. Esse movimento revela padrões que atravessam diferentes narrativas e que não se distribuem de maneira homogênea^(1,6,22)^. Processos de subcuidado, suspeição moral ou deslegitimação da narrativa tendem a incidir com maior intensidade sobre mulheres situadas em intersecções específicas de raça, gênero e classe^(24,25,26,27)^. A comparação constante permite conectar essas regularidades às estruturas institucionais que organizam o cuidado, deslocando a análise do plano exclusivamente interpessoal para a compreensão de mecanismos estruturais.

A codificação teórica integra essas categorias em um modelo explicativo^(3,11,28)^ que torna evidente como discursos biomédicos, protocolos assistenciais e hierarquias sociais operam conjuntamente na produção do racismo obstétrico^(26)^. A interseccionalidade atua, nesse processo, como lente crítica que explicita a imbricação entre racismo, sexismo e classismo, enquanto a TFC funciona como tecnologia analítica que organiza esses elementos em teoria substantiva. A análise deixa de ser apenas descritiva e passa a modelar os regimes institucionais que sustentam o subcuidado.

Em todas as etapas, a reflexividade ocupa lugar central^(3,28,29)^. A TFC exige que o(a) pesquisador(a) reconheça sua posição social, seus pressupostos e suas implicações ético-políticas na construção do conhecimento^(2,23)^. Na investigação do racismo obstétrico, essa vigilância epistemológica é indispensável para evitar a naturalização das desigualdades ou a reprodução de discursos institucionais que minimizam a violência racial. As categorias emergentes são compreendidas como construções interpretativas, ancoradas em contextos históricos de opressão e resistência.

Assim, ao localizar as ações e os significados produzidos pelas mulheres em suas narrativas, o(a) pesquisador(a) revela as conexões entre os níveis micro e macro da análise. No plano subjetivo, o que a mulher sente, interpreta ou nomeia como injustiça passa a ser articulado ao plano estrutural, aos protocolos, aos discursos e às práticas institucionais que organizam o cuidado. Nesse sentido, a TFC não se limita a descrever experiências, mas busca analisar as suposições implícitas que sustentam as ações e interpretações dos(as) participantes. Para Charmaz, a análise constitui uma representação interpretativa da realidade estudada, não um relatório objetivo ou uma descrição definitiva do fenômeno^(2,3,6,23)^. Tal compreensão aguça a consciência do(a) pesquisador(a) para a relatividade dos dados e para a existência de múltiplas realidades coexistentes, condição essencial para uma abordagem interseccional.

Desse modo, a integração entre TFC e interseccionalidade não apenas descreve experiências de injustiça reprodutiva, mas modela os regimes institucionais que as produzem. Ao transformar a lente interseccional em procedimento analítico concreto, a TFC possibilita articular níveis micro e macro de análise e construir uma teoria substantiva comprometida com a explicitação e o enfrentamento das desigualdades racializadas no cuidado obstétrico.

## CONSIDERAÇÕES FINAIS

Como parte da compreensão trabalhada ao longo do texto, pode-se inferir que a TFD, em sua vertente construtivista, e o referencial teórico da interseccionalidade podem ser articulados de forma complementar para analisar fenômenos sociais complexos, especialmente aqueles marcados por desigualdades estruturais. Ambas as abordagens compartilham do mesmo compromisso com a escuta atenta das experiências vividas, com o reconhecimento das múltiplas dimensões da opressão e com a produção de conhecimento comprometido com a transformação social. Essa articulação metodológica-teórica permite que a pesquisa não apenas apresente o fenômeno investigado, mas também contribua para a promoção da equidade e da justiça social.

A TFC parte do princípio de que os significados sociais são construídos a partir das interações e dos contextos socioculturais. Ao valorizar preceitos da TFC, como a reflexividade do(a) pesquisador(a), o diálogo com os(as) participantes e a coconstrução dos significados, cria-se um ambiente propício para a aplicação da interseccionalidade. Isso porque o(a) pesquisador(a) não se posiciona como sujeito neutro ou externo, mas reconhece o seu lugar social e a sua implicação nas relações de poder que a própria pesquisa busca problematizar.

Portanto, ao integrar a TFC e a interseccionalidade, o(a) pesquisador(a) assume uma postura ética e política, comprometida com a valorização das vozes historicamente silenciadas e com a produção de conhecimento transformador. Essa aliança entre teoria e método possibilita que a pesquisa vá além da denúncia, oferecendo subsídios para a formulação de práticas e políticas mais justas e inclusivas no campo da saúde reprodutiva. Desse modo, trata-se de uma estratégia investigativa que dialoga diretamente com os princípios da justiça social, ao reconhecer, compreender e enfrentar as múltiplas formas de opressão que estruturam a vida de mulheres e pessoas com útero em situação de vulnerabilidade.

## Data Availability

Todo o conjunto de dados que dá suporte aos resultados deste estudo foi publicado no próprio artigo.

# Translation: Teoría fundamentada constructivista e interseccionalidad en la investigación cualitativa crítica: caminos para comprender la injusticia reproductiva

**DOI:** 10.1590/1980-220X-REEUSP-2025-0331es

**Authors:** Ariane Teixeira de Santana, Telmara Menezes Couto, Patricia Santos de Oliveira

**Affiliations:** 1 Universidade Federal da Bahia, Escola de Enfermagem, Salvador, BA, Brasil.

**Keywords:** Teoría Fundamentada, Marco Interseccional, Teoría Crítica, Investigación Cualitativa, Métodos

## Abstract

**Objetivo:**

Reflexionar sobre el potencial de la Teoría Fundamentada Constructivista, articulada con la interseccionalidad, como estrategia metodológica y epistemológica crítica para la investigación de las injusticias reproductivas.

**Método:**

Estudio teórico y reflexivo desde una perspectiva dialéctica, utilizando la investigación bibliográfica como recurso.

**Resultados:**

La articulación entre el enfoque constructivista de la Teoría Fundamentada Constructivista y la interseccionalidad permite comprender fenómenos como el racismo obstétrico a partir de la coconstrucción de significados, evidenciada por cómo la raza, el género y la clase operan relacionalmente en la producción de desigualdades en la atención reproductiva. Esta integración metodológica-teórica amplía el alcance analítico de la investigación cualitativa al incorporar la reflexividad del investigador.

**Consideraciones finales:**

La combinación de estos enfoques fortalece la producción de conocimiento comprometido con la transformación social, brindando apoyo a las investigaciones críticas en enfermería y salud pública. Su aplicabilidad radica en la posibilidad de orientar la investigación sensible a las desigualdades estructurales, contribuyendo a la formulación de prácticas y políticas de atención reproductiva más equitativas.

## INTRODUCCIÓN

La Teoría Fundamentada en los Datos (TFD) se presenta como una opción metodológica en la investigación cualitativa, combinando directrices para la recolección y el análisis de datos. Se trata de un enfoque inductivo, en el que las nuevas teorías sobre los procesos sociales se basan en experiencias reales. Desde su concepción, la TFD se ha convertido en una herramienta muy poderosa para las ciencias sociales^(1,2)^.

La TFD fue desarrollada por dos sociólogos, Barney Glaser y Anselm Strauss, en los Estados Unidos a principios de la década de 1960. Inicialmente, Glaser y Strauss colaboraron en un proyecto de investigación sobre la interacción entre pacientes y profesionales de la salud en hospitales, y sus ideas se publicaron por primera vez en *Discovery of Grounded Theory*, en 1967. Durante ese proyecto, desarrollaron una metodología de investigación que se convirtió en la base de la TFD^(1,3)^. Es evidente que el método no surgió únicamente del trabajo de Glaser y Strauss. Si observamos los trabajos anteriores desarrollados en la Escuela de Chicago, veremos elementos en común con otros métodos^(1)^.

Aunque Glaser y Strauss contribuyeron inicialmente al desarrollo de la TFD, con el tiempo desarrollaron vertientes diferentes para la aplicación de la metodología^(3)^. Estos enfoques presentan diferentes supuestos filosóficos que influyen en las formas en que se comprenden e implementan sus métodos, pero conservan similitudes fundamentales importantes. Comprender las similitudes y diferencias presentes en estos enfoques puede facilitar el recorrido metodológico y determinar la mejor adecuación al estudio y a la visión del mundo de cada investigador^(4)^.

De este modo, el enfoque constructivista, desarrollado por la socióloga Kathy Charmaz, quien reposicionó la TFD hacia tal postura con la ontología relativista y la epistemología subjetiva, se define como el que mejor se ajusta al presente estudio, que trata sobre la injusticia reproductiva. La Teoría Fundada Constructivista (TFC) tiene una epistemología subjetiva, pues asume que los investigadores no están separados de la investigación y que el conocimiento se co-crea^(3)^.

La TFC valora la experiencia subjetiva y el significado que los individuos atribuyen a sus vivencias. En contextos en los que la atención de salud refleja desigualdades sociales, esta vertiente ofrece un enfoque esencialmente humanizador. En lugar de tratar las experiencias individuales como datos “objetivos”, el investigador constructivista debe comprender estas vivencias como reflejos de relaciones sociales, culturales e institucionales^(2)^. En el contexto de la justicia reproductiva, esta perspectiva es fundamental, ya que ayuda a considerar que la atención obstétrica no ocurre en un vacío, sino dentro de una red de normas sociales y estructurales que afectan de manera desigual a las personas.

Como lente teórica, la interseccionalidad propuesta por Kimberlé Crenshaw ofrece un marco para comprender cómo diferentes formas de opresión, como el racismo, el sexismo y el clasismo, interactúan y moldean las experiencias de las personas marginadas^(5)^. La salud reproductiva y la atención obstétrica son áreas en las que estas opresiones se manifiestan con frecuencia de manera interseccional. Las mujeres negras y pobres, por ejemplo, enfrentan no solo el sexismo inherente a la negación de sus decisiones reproductivas, sino también el racismo que las clasifica, a menudo, como “menos merecedoras” de una atención respetuosa y calificada.

Ante estas consideraciones, este ensayo se propone reflexionar sobre cómo la TFC puede utilizarse como herramienta metodológica, a partir del bagaje analítico teórico interseccional. De este modo, con el fin de contribuir críticamente, este texto tiene como objetivo reflexionar sobre el potencial de la TFC articulada con la interseccionalidad como herramienta metodológica y epistemológica crítica en la investigación de las injusticias reproductivas.

## MÉTODO

Se trata de un estudio teórico de naturaleza crítico-reflexiva, fundamentado en el análisis conceptual y la problematización epistemológica de producciones científicas nacionales e internacionales sobre la reflexión dialéctica entre la TFC y la interseccionalidad en la investigación de las injusticias reproductivas.

La producción teórica se construyó a partir de la lectura sistemática de obras clásicas y contemporáneas, mediante una revisión bibliográfica en libros y en bases de datos nacionales e internacionales, como Scopus, *Web of Science*, PubMed y SciELO. Se incluyeron publicaciones que discuten los fundamentos epistemológicos y metodológicos de la TFC, producciones teóricas y empíricas sobre la interseccionalidad como herramienta analítica crítica, estudios que articulan la investigación cualitativa, la justicia social y la salud, y literatura relacionada con el racismo obstétrico y las desigualdades reproductivas.

El análisis se desarrolló mediante una lectura crítica y comparativa de las obras seleccionadas, la identificación de convergencias y tensiones conceptuales y la construcción de síntesis interpretativas orientadas por las preguntas analíticas: ¿cómo contribuye este enfoque a la comprensión de las injusticias reproductivas? ¿De qué manera la articulación entre la TFC y la interseccionalidad amplía el potencial crítico de la investigación cualitativa?

El marco epistemológico que sustenta este ensayo se basa en la ontología relativista y la epistemología subjetiva, propias de la vertiente constructivista de la TFD, que conciben el conocimiento como una construcción social situada, producida en la interacción entre el investigador, los participantes y el contexto^(2,3,6)^. A esta postura se suma la interseccionalidad como teoría social crítica, que reconoce la imbricación entre múltiples sistemas de poder, como el racismo, el patriarcado y el clasismo, en la producción de desigualdades estructurales^(7,8)^.

Esta estrategia metodológica no solo permitió la descripción de los marcos de referencia analizados, sino también su interpretación crítica, revelando sus contribuciones, límites y posibilidades de aplicación en el campo de la investigación en salud reproductiva y la lucha contra el racismo obstétrico.

### La Teoría Fundamentada Constructivista y sus Supuestos

Kathleen Marian Charmaz, nacida el 19 de agosto de 1939 en Whitehall, Wisconsin, falleció el 27 de julio de 2020. Fue una socióloga estadounidense de la segunda generación de teóricos fundamentados. Kathy Charmaz se convirtió en una innovadora y reconocida teórica activa en el enfoque metodológico cualitativo, desarrollando el método de la TFC.

Charmaz señala que el investigador no es un observador neutral, sino un participante activo en la construcción del conocimiento, influenciado por sus valores y experiencias, a diferencia de la visión clásica y objetivista de Glaser y Strauss^(3,9)^.

Las bases epistemológicas y ontológicas de la TFD han cambiado a lo largo de los años, especialmente con los desafíos planteados por la corriente constructivista^(1)^. La TFC representa un enfoque cualitativo externo para la construcción de una teoría que surge de los datos de investigación en interacción con el investigador. Es importante destacar, sin embargo, que a pesar de ser una revisión contemporánea, conserva los fundamentos pragmáticos de la TFD propuesta por Glaser y Strauss^(10,11)^.

La posición constructivista asumida por Charmaz presenta una ontología relativista y una epistemología subjetiva, y esto explica cómo la producción de conocimiento es un elemento socialmente construido y reconocido desde múltiples puntos de vista, tanto del participante en la investigación como del investigador. Propone una postura reflexiva en relación con los datos presentados en la interacción del investigador con los participantes en el escenario de campo para las construcciones analíticas^(3)^. Se observa, así, la localidad humana del investigador o investigadora en la producción de la investigación y cómo el proceso y el producto se generan a partir de esas intersecciones.

Los investigadores que utilizan la TFC pueden guiarse por diversos puntos de partida teóricos para iniciar la investigación, como la teoría feminista, el posestructuralismo, la teoría marxista, las teorías críticas o el propio Interaccionismo Simbólico. Según Charmaz, aunque el Interaccionismo Simbólico y la TFD forman un poderoso “paquete teoría-métodos”, el investigador o investigadora fundamentado no debe suscribir una ortodoxia entre el teórico interaccionismo simbólico y la TFD. Por lo tanto, es importante comprender que la TFD puede utilizarse a partir de otros puntos de partida teóricos o repertorios analíticos para respaldar los datos^(3,12)^.

Para Charmaz, el conocimiento se basa en construcciones sociales. Los estudiosos que utilizan la TFC deben comprender que el conocimiento se produce al lidiar con problemas empíricos. La construcción del conocimiento ocurre bajo condiciones estructurales preexistentes y situaciones emergentes, y se ve influida por las perspectivas, los privilegios, las posiciones sociales, las interacciones y las ubicaciones geográficas del investigador o la investigadora. Todas estas condiciones son inherentes a la situación de investigación, pero son completamente ignoradas o no mencionadas por los estudios^(3,13)^.

La TFC valora la complejidad y la multiplicidad de significados que se presentan en los datos, ya que es a partir de ellos que se construye una comprensión más rica de las características sociales. Un ejemplo es la idea de que las realidades sociales y las interpretaciones construidas no son neutras, sino que están moldeadas por los contextos históricos, culturales y sociales, tanto de los participantes como de los investigadores^(1,13)^.

Al localizar los significados y las acciones de los participantes, el investigador revela las conexiones entre los niveles micro y macro de análisis, lo que vincula, en este contexto, el plano subjetivo y el social del objeto estudiado. Los significados de los participantes pueden reflejar ideologías, acciones, convenciones sociales o relaciones de poder. La TFC busca analizar las suposiciones a partir de las cuales los participantes construyen sus significados y acciones. Charmaz veía el análisis en la TFC como una representación interpretativa, no como informes objetivos o un único punto de vista sobre el tema. Como resultado, el investigador agudiza la conciencia sobre la relatividad de los datos, que presentan sus múltiples realidades^(1,3)^.

El proceso de codificación de la TFC es similar al de la Teoría Fundada Clásica, ya que cuenta con al menos dos fases, la codificación inicial y la focalizada, y se presenta menos prescriptiva en su análisis de datos. La codificación inicial es la primera fase del análisis; en ella, el investigador explora los datos de manera detallada, línea por línea, incidente por incidente, para identificar los códigos iniciales. En esta etapa, los códigos se crean a partir de lo que está sucediendo en cada segmento de los datos, lo que permite un análisis abierto y flexible^(3,10)^. Para esta etapa, se recomienda que los investigadores utilicen verbos en gerundio para los códigos iniciales, ya que esto ayuda a captar la acción, el proceso o el significado contenido en los discursos de los participantes. En lugar de etiquetar la experiencia directamente, el investigador intenta interpretar lo que está sucediendo en los discursos, observaciones o textos analizados. Estos códigos iniciales suelen ser variados y pueden ajustarse y refinarse durante el análisis^(3)^.

En la segunda fase, la codificación focalizada, el investigador revisa los códigos iniciales y selecciona aquellos que son más significativos o recurrentes en los datos para generar categorías provisionales. La codificación focalizada ayuda a simplificar y organizar los datos de manera más agrupada, lo que contribuye a la identificación de categorías y patrones más amplios^(3)^. Esta fase implica un nivel más alto de interpretación, ya que el investigador o investigadora comienza a comparar los códigos iniciales entre sí y a agrupar los datos en categorías emergentes. El objetivo es identificar códigos que sean más representativos de los aspectos centrales para explicar los patrones en los datos. De este modo, se refinan los conceptos en relación con los datos de una manera más abstracta, lo que permite una búsqueda para comprender las conexiones y estructuras subyacentes^(3)^.

La codificación teórica comienza con la codificación focalizada y culmina en la teoría final, con el objetivo de integrar las categorías y generar una teoría sustantiva. En esta etapa, el investigador o investigadora utiliza los códigos focalizados para desarrollar una comprensión más amplia, a fin de identificar relaciones e interacciones entre las categorías principales. La codificación teórica ayuda a construir una narrativa teórica que explique los procesos y patrones observados en los datos^(3)^. La TFC enfatiza que la convicción teórica es un proceso interpretativo en el que el investigador busca construir una teoría que sea fiel al contexto y a las experiencias de los participantes. La fase teórica da como resultado un análisis más completo y profundo, que conecta y da sentido a diferentes aspectos de los datos^(11)^.

En todas las fases de codificación de la TFC, la reflexividad es importante. El investigador debe ser continuamente consciente de su propia posición, influencias e interpretaciones. Esto significa considerar su papel en la construcción del conocimiento al percibir que los códigos y las categorías son resultados de una interpretación activa, y no una descripción neutral u objetiva de los datos^(3)^.

Otro aspecto importante en el TFC es el uso de memorandos (memos) y diagramas para respaldar el proceso de consolidación y ayudar en el desarrollo teórico. Los memorandos son anotaciones reflexivas que documentan ideas, cuestionamientos y *insights* durante la realización de la investigación, lo que permite al investigador explorar y reflexionar sobre las interpretaciones que surgen de los datos. Los diagramas, por su parte, ayudan a visualizar las relaciones entre las categorías y facilitan la organización de las ideas y la construcción de la teoría^(3,10,11,12)^.

El TFC describe, de manera precisa, cómo se da el pensamiento inductivo y abductivo del investigador, siendo esta una etapa interactiva del análisis que requerirá del analista un razonamiento que permita ir y venir entre los datos y la conceptualización como parte fundamental del análisis de datos^(3)^.

### La Interseccionalidad como Herramienta de Análisis Crítico

En la década de 1990, la investigadora y activista Kimberlé Crenshaw, junto con otras investigadoras, definió el término “interseccionalidad” para designar las intersecciones entre raza, género, clase y otros ejes de subordinación^(5)^.

Para Crenshaw, la interseccionalidad busca conjugar las consecuencias estructurales y dinámicas de la interacción entre dos o más ejes de subordinación al analizar la interacción entre el racismo, el patriarcado, la explotación de clase y otros sistemas de opresión, exponiendo las desigualdades constitutivas de la sociedad^(5,14,15,16)^.

Según las teóricas del feminismo negro, la interseccionalidad ayuda a comprender la articulación de diferentes categorías sociales que se entrelazan y estructuran la vida de las personas en la sociedad, especialmente de las mujeres negras. Esta subalternización pone de manifiesto la opresión, la discriminación, la desigualdad y la injusticia social que estas personas experimentan. La interseccionalidad facilita la visión de las interrelaciones que generan estereotipos y jerarquizan a las personas dentro de las construcciones sociales de poder^(5,16)^.

En Brasil, las raíces de la construcción de la interseccionalidad como teoría están vinculadas a movimientos sociales y académicos que cobraron importancia a partir de la década de 1990. El movimiento negro desempeñó un papel fundamental en esta producción, al destacar las interacciones entre raza, clase y género en la experiencia de las mujeres negras y las personas marginadas. El pensamiento feminista negro brasileño, liderado por figuras como Lélia Gonzalez y Sueli Carneiro, fue un gran impulsor del pensamiento interseccional. Estas intelectuales señalaban, de manera interseccional, consideraciones sobre los problemas que enfrenta la población negra, especialmente las mujeres(17,18).

De este modo, tanto las intelectuales y activistas brasileñas como las estadounidenses se encargaron de denunciar la insuficiencia de un feminismo hegemónico que, al universalizar la experiencia de las mujeres blancas de clase media, silenciaba las voces de las mujeres negras y marginadas. Paralelamente, también criticaban al movimiento negro por no reconocer las especificidades de las opresiones de género vividas por las mujeres negras. A partir de estas tensiones, el feminismo negro construyó un cuerpo teórico y político propio, que buscaba comprender las interacciones entre diferentes sistemas de dominación^(15,17)^.

Para una comprensión más adecuada de los problemas sociales complejos, se forja la interseccionalidad. En el ámbito de la justicia social, se propuso identificar la discriminación racial y la discriminación de género para comprender más profundamente cómo estas discriminaciones operan juntas e imponen limitaciones a las mujeres negras^(16)^.

Crenshaw, en 1991, escribió el artículo “*Mapping the Margins: Intersectionality, Identity Politics, and Violence against Women of Color*”, y en él utiliza el término para analizar la posición interseccional de las mujeres negras y su marginación estructural^(15)^. A partir de este trabajo, la autora concibe una base teórica sólida para comprender la interseccionalidad como una herramienta de análisis y *práctica* crítica, entretejiendo aportes de la teoría crítica de la raza y estableciendo el concepto provisional de “interseccionalidad”. Esta importante obra contribuyó al desarrollo de la teoría como un enfoque crucial tanto en los movimientos sociales como en la investigación académica sobre raza, género y clase^(5,8)^.

Según el pensamiento de Crenshaw, los estudios que buscan comprender a las mujeres negras no pueden utilizar una única categoría de análisis. Para ella, estas experiencias solo pueden entenderse cuando se examinan las múltiples identidades sociales que estas mujeres llevan consigo. Así, a partir de esta lente crítica, podremos ver cómo los múltiples sistemas de poder son intrínsecos a la experiencia social de estas mujeres. La interseccionalidad es una base analítica que describe cómo los sistemas de poder se construyen mutuamente y producen lugares sociales distintos para cada individuo y grupo dentro de ellos^(5)^.

En la construcción conceptual de la interseccionalidad, Crenshaw señala los peligros de la invisibilidad analítica que surgen cuando una mirada se concentra exclusivamente en un solo marcador social, descuidando los demás^(5,14)^. Con miras a esta comprensión, presentó los conceptos de superinclusión y subinclusión, en un intento por esclarecer esta invisibilidad. Según la autora, los enfoques que consideran solo un eje de desigualdad, ya sea la raza o el género, tienden a camuflar las experiencias de aquellas que se encuentran en la intersección entre diferentes sistemas de opresión.

La interseccionalidad, como herramienta de análisis crítico, no se limita a reconocer únicamente las opresiones que imponen las múltiples identidades sociales, sino que se propone valorar las estrategias de resistencia y empoderamiento que se configuran a partir de las intersecciones de esas identidades. Esto es fundamental para desarrollar estrategias de lucha contra la opresión y promover la justicia social^(7,8,16,19)^.

Para comprender cómo se producen estas interacciones, Collins desarrolló el concepto de “matriz de dominación”. Este ayuda a analizar las intersecciones entre la estructura de poder de una sociedad.

Para ella, la matriz es una estructura multidimensional compuesta por diversas formas de opresión y discriminación que se entrelazan y se refuerzan mutuamente. Estas formas de opresión incluyen, entre otras, el racismo, el sexismo, el clasismo y la homofobia^(5,7)^.

Como teoría social crítica, la interseccionalidad es una herramienta contrahegemónica que se centra en la construcción de un análisis teórico crítico al examinar, a partir de sus supuestos, la comprensión de las experiencias de las personas marginadas y al destacar las voces y los conocimientos de estos individuos para su emancipación social. Como práctica reflexiva, la interseccionalidad busca transformar las estructuras de poder existentes. Al utilizar un enfoque interseccional, el/la investigador/a debe construir un análisis que exponga las estructuras de poder dominantes, además de buscar promover una mayor inclusión y equidad en la sociedad^(7)^.

En este sentido, la interseccionalidad se consolida políticamente en el ámbito de las perspectivas críticas de las ciencias sociales, particularmente en diálogo con los estudios feministas, antirracistas y decoloniales. A partir de estos diálogos teóricos, se afirma como una teoría social crítica capaz de revelar las interconexiones entre diferentes sistemas de poder. En el plano epistémico, la interseccionalidad se configura como una herramienta analítica fundamental para comprender los procesos históricos e institucionales que producen y reproducen las desigualdades sociales.

### Teoría Fundamentada Constructivista en la Investigación Crítica en la Investigación de Justicia Social

La TFC, según los preceptos de Charmaz, se desarrolla como un campo de investigación crítica al romper con los enfoques positivistas y asumir que el conocimiento es construido, situado e influenciado por las relaciones de poder. Este enfoque amplía el alcance de la TFD^(2)^, permitiendo que se utilice como herramienta de cuestionamiento y transformación social, especialmente en contextos marcados por las desigualdades, como en el racismo obstétrico, la violencia de género y las injusticias reproductivas.

La TFC parte de la comprensión de que la realidad y el conocimiento son construcciones sociales. Esto significa que los constructos surgen de la interacción entre investigadores y participantes, en contextos específicos, y que no hay una “verdad objetiva” por descubrir. Esta postura epistemológica favorece una investigación crítica al permitir analizar cómo los discursos y las prácticas producen y reproducen desigualdades sociales^(2,3)^.

En los contextos en los que la opresión, la discriminación y la desigualdad se han convertido en el *status quo,* es necesario cuestionar las causas. En este sentido, Charmaz orienta el énfasis en el uso de preguntas estratégicas como “¿Por qué?” y “¿Qué está pasando aquí?”. Estas preguntas ayudan al investigador o investigadora a realizar una evaluación más crítica, al situar a los participantes de la investigación dentro de las condiciones sociales, culturales, temporales y situacionales en las que viven. Esto ayuda a reconocer cómo las condiciones y posiciones estructurales afectan al investigador o investigadora y al proceso de investigación^(2,7)^.

En la TFC, se invita a los investigadores a situarse en lo que está sucediendo en el espacio de la investigación, reconociéndose como parte de ella y permaneciendo flexibles y atentos a los eventos empíricos, al lenguaje y al significado, asumiendo, sobre todo, las responsabilidades morales derivadas de la investigación. Este enfoque exige un examen minucioso de cómo los descubrimientos posteriores se relacionan con estas condiciones. Con énfasis en la reflexividad, no solo se examina quién se es en relación con la investigación, sino también cómo se establece esta dialéctica^(2,20,21,22)^.

El investigador o la investigadora en la TFC no es neutral. Es parte de la construcción del conocimiento y, por ello, es necesario adoptar una postura reflexiva a partir de las siguientes preguntas: ¿quién soy? ¿Cuál es mi lugar social? ¿Cómo afectan mis creencias y experiencias a la Recolección y el análisis de datos? Esta reflexividad crítica fortalece el rigor de la investigación y amplía el compromiso ético y político^(2,3)^.

La perspectiva constructivista entiende la investigación como un proceso político y ético. El objetivo no es solo comprender, sino también intervenir en las realidades injustas. Al construir teorías a partir de la experiencia vivida de grupos históricamente marginados (como mujeres negras, indígenas, personas trans), la TFC revela mecanismos de opresión y abre espacio para la resistencia^(2)^.

Una de las grandes ventajas de la TFC es dar centralidad a las experiencias y narrativas de los sujetos oprimidos. Esto es especialmente importante al estudiar temas como el racismo obstétrico, en el que los silencios institucionales y los discursos hegemónicos intentan negar o minimizar el problema. Al escuchar atentamente y con sensibilidad crítica las voces silenciadas, la TFC adopta la epistemología del pensamiento crítico.

El enfoque constructivista está atento a las relaciones de poder que atraviesan el proceso de investigación, tanto en lo que respecta a las experiencias de los participantes como a la postura de los investigadores. Esto permite una lectura crítica de las estructuras institucionales, culturales y simbólicas que sustentan las prácticas discriminatorias^(2,3)^. La TFC, cuando se asocia a marcos de referencia como la interseccionalidad, ayuda a revelar cómo la clase, la raza, el género y otras categorías interactúan para producir desigualdades.

Según Charmaz, como investigación crítica, la TFC permite acompañar la complejidad de los fenómenos sociales sin reducirlos a categorías cerradas. En lugar de “encajar” los datos en teorías preexistentes, busca crear teorías sensibles, contextuales y dinámicas, construidas a partir de los significados de los participantes^(2)^. Esta apertura es esencial para captar los efectos del racismo institucional, de las políticas públicas excluyentes y de la negligencia con los derechos reproductivos.

Las investigaciones que utilizan la TFC con un enfoque en la justicia social se desarrollan a partir de la ética relacional, reconociendo que la relación con los participantes es dialógica, respetuosa y horizontal. La investigación no se realiza “sobre” los sujetos, sino con ellos. Este posicionamiento ético fortalece el compromiso con cambios que beneficien a los grupos involucrados. Esta postura favorece la identificación, el análisis y el cuestionamiento de las estructuras institucionales, culturales y simbólicas que mantienen las desigualdades sociales, poniendo de manifiesto cómo los sistemas de salud, educación, justicia o asistencia social pueden reproducir el racismo, el sexismo, el clasismo y otros prejuicios^(20,21,22)^.

La TFC se basa en su herencia pragmática de abordar problemas prácticos y promover la justicia social^(23)^, al enfatizar el diseño de procesos y promover la creación de conexiones entre eventos y situaciones, significados y acciones, individuos y estructuras sociales que pueden permanecer invisibles.

Esta versión de la TFD proporciona herramientas que permiten a los investigadores profundizar en las situaciones estudiadas y les brinda la oportunidad de analizarlas desde diversos puntos de vista. Así, al conceptualizar lo aprendido, los teóricos constructivistas fundamentados pueden ofrecer nuevos entendimientos críticos. Para Charmaz, la TFC invita a examinar no solo las decisiones de investigación, sino también el compromiso moral y social con lo que se estudia^(2)^.

### Teoría Fundamentada Constructivista y Análisis Interseccional en la Investigación del Racismo Obstétrico

Las investigaciones que utilizan la TFC desde una perspectiva crítica hacen referencia a un corpus específico de conocimiento que cuestiona los efectos opresivos del poder institucional, del conocimiento y de los problemas sociales^(21,23)^. La TFC, en un enfoque crítico, amplía la TFC, integrando métodos participativos e incorporando objetivos de cambio social transformador^(20)^.

Este enfoque representa una metodología sensible a la complejidad de los fenómenos sociales. Al romper con el positivismo original de la TFD, reconoce las dimensiones subjetivas y contextuales del proceso de investigación^(2,3)^. Esta perspectiva es especialmente relevante para investigar experiencias marcadas por desigualdades estructurales, como es el caso del racismo obstétrico, una forma específica de violencia que incide sobre cuerpos socialmente racializados en el ámbito de la salud reproductiva.

Investigaciones recientes han demostrado disparidades raciales persistentes en los resultados maternos y en la calidad de la atención obstétrica, lo que evidencia una mayor exposición de las mujeres negras a una atención precaria^(24,25,26,27)^. Sin embargo, una parte significativa de estos estudios sigue centrada en la descripción de las desigualdades o en la identificación de factores aislados, sin modelar procesalmente cómo se producen tales disparidades en las interacciones institucionales cotidianas^(24,25,27)^. La articulación entre la TFC y la interseccionalidad ofrece un camino metodológico capaz de llenar esta brecha al transformar la problematización de las desigualdades en una construcción teórica sistemática.

La TFC parte del supuesto de que la realidad social es múltiple, relacional e históricamente situada^(2,28,29)^. Al valorar la complejidad y la multiplicidad de significados presentes en los datos, la TFC reconoce que las interpretaciones no son neutras, sino que están moldeadas por contextos históricos, culturales y sociales que atraviesan tanto a los participantes como a los investigadores^(3,11,22,23)^.

En el caso del racismo obstétrico, las experiencias relatadas por las mujeres no surgen en un vacío social. Están inmersas en tradiciones institucionales, ideologías biomédicas, jerarquías raciales y normas de género que estructuran el campo de la salud reproductiva. Esto indica que la TFC y la interseccionalidad ofrecen un camino metodológico capaz de traducir este escenario.

La operacionalización de la interseccionalidad en la TFC se lleva a cabo mediante la codificación procesual. En esta etapa, el/la investigador/a examina el material línea por línea para captar acciones e interacciones. Esta estrategia evita clasificaciones prematuras y permite observar cómo la raza, el género y la clase se articulan dinámicamente en la experiencia de la atención^(3,11,23,30)^. Al privilegiar los procesos, el análisis impide la fijación de identidades como categorías estáticas y pone de manifiesto la producción social de las desigualdades.

En la codificación focalizada, los códigos iniciales se comparan y agrupan en categorías más densas y explicativas^(3,11)^. Este movimiento revela patrones que atraviesan diferentes narrativas y que no se distribuyen de manera homogénea^(1,6,22)^. Los procesos de subatención, sospecha moral o deslegitimación de la narrativa tienden a incidir con mayor intensidad sobre las mujeres situadas en intersecciones específicas de raza, género y clase^(24,25,26,27)^. La comparación constante permite conectar estas regularidades con las estructuras institucionales que organizan la atención, desplazando el análisis del plano exclusivamente interpersonal hacia la comprensión de los mecanismos estructurales.

La codificación teórica integra estas categorías en un modelo explicativo^(3,11,28)^ que pone de manifiesto cómo los discursos biomédicos, los protocolos asistenciales y las jerarquías sociales operan conjuntamente en la producción del racismo obstétrico^(26)^. La interseccionalidad actúa, en este proceso, como una lente crítica que explicita la imbricación entre racismo, sexismo y clasismo, mientras que la TFC funciona como una tecnología analítica que organiza estos elementos en una teoría sustantiva. El análisis deja de ser meramente descriptivo y pasa a modelar los regímenes institucionales que sustentan la falta de atención.

En todas las etapas, la reflexividad ocupa un lugar central^(3,28,29)^. La TFC exige que el/la investigador/a reconozca su posición social, sus supuestos y sus implicaciones ético-políticas en la construcción del conocimiento^(2,23)^. En la investigación del racismo obstétrico, esta vigilancia epistemológica es indispensable para evitar la naturalización de las desigualdades o la reproducción de discursos institucionales que minimizan la violencia racial. Las categorías emergentes se entienden como construcciones interpretativas, ancladas en contextos históricos de opresión y resistencia.

Así, al ubicar las acciones y los significados producidos por las mujeres en sus narrativas, el/la investigador/a revela las conexiones entre los niveles micro y macro del análisis. En el plano subjetivo, lo que la mujer siente, interpreta o denomina como injusticia pasa a articularse con el plano estructural, los protocolos, los discursos y las prácticas institucionales que organizan el cuidado. En este sentido, el TFC no se limita a describir experiencias, sino que busca analizar las suposiciones implícitas que sustentan las acciones e interpretaciones de los participantes. Para Charmaz, el análisis constituye una representación interpretativa de la realidad estudiada, no un informe objetivo o una descripción definitiva del fenómeno^(2,3,6,23)^. Tal comprensión agudiza la conciencia del investigador o investigadora respecto a la relatividad de los datos y a la existencia de múltiples realidades coexistentes, condición esencial para un enfoque interseccional.

De este modo, la integración entre la TFC y la interseccionalidad no solo describe experiencias de injusticia reproductiva, sino que modela los regímenes institucionales que las producen. Al transformar la lente interseccional en un procedimiento analítico concreto, la TFC permite articular niveles micro y macro de análisis y construir una teoría sustantiva comprometida con la explicitación y el enfrentamiento de las desigualdades racializadas en la atención obstétrica.

## CONSIDERACIONES FINALES

Como parte de la comprensión desarrollada a lo largo del texto, se puede inferir que la TFD, en su vertiente constructivista, y el marco teórico de la interseccionalidad pueden articularse de manera complementaria para analizar fenómenos sociales complejos, especialmente aquellos marcados por desigualdades estructurales. Ambos enfoques comparten el mismo compromiso con la escucha atenta de las experiencias vividas, con el reconocimiento de las múltiples dimensiones de la opresión y con la producción de conocimiento comprometido con la transformación social. Esta articulación metodológico-teórica permite que la investigación no solo presente el fenómeno investigado, sino que también contribuya a la promoción de la equidad y la justicia social.

La TFC parte del principio de que los significados sociales se construyen a partir de las interacciones y los contextos socioculturales. Al valorar los preceptos de la TFC, como la reflexividad del investigador o investigadora, el diálogo con los participantes y la co-construcción de significados, se crea un ambiente propicio para la aplicación de la interseccionalidad. Esto se debe a que el/la investigador/a no se posiciona como un sujeto neutral o externo, sino que reconoce su lugar social y su implicación en las relaciones de poder que la propia investigación busca problematizar.

Por lo tanto, al integrar la TFC y la interseccionalidad, el/la investigador/a (a) asume una postura ética y política, comprometida con la valorización de las voces históricamente silenciadas y con la producción de conocimiento transformador. Esta alianza entre teoría y método permite que la investigación vaya más allá de la denuncia, ofreciendo insumos para la formulación de prácticas y políticas más justas e inclusivas en el campo de la salud reproductiva. De este modo, se trata de una estrategia investigativa que dialoga directamente con los principios de la justicia social, al reconocer, comprender y enfrentar las múltiples formas de opresión que estructuran la vida de las mujeres y las personas con útero en situación de vulnerabilidad.

## Data Availability

Todo el conjunto de datos que apoya los resultados de este estudio fue publicado en el propio artículo.
